# Reducing rehospitalization in cardiac patients: a randomized, controlled trial of a cardiac care management program (“Cardiolotse”) in Germany

**DOI:** 10.1186/s12916-024-03691-7

**Published:** 2024-10-21

**Authors:** Wiebke Schüttig, Harald Darius, Katrin C. Reber, Marie Coors, Amelie Flothow, Alfred Holzgreve, Sebastian Karmann, Anica Stürtz, Rebecca Zöller, Saskia Kropp, Petra Riesner, Leonie Sundmacher

**Affiliations:** 1 Chair of Health Economics, TUM School of Medicine and Health, Technical University of Munich, Georg-Brauchle-Ring 60/62, Munich, 80992 Germany; 2 AOK Nordost - Die Gesundheitskasse, Health Services Management, Berlin, Germany; 3 Vivantes – Netzwerk für Gesundheit GmbH, Berlin, Germany

**Keywords:** Discharge, Cardiovascular disease, Care management, Socioeconomics, Hospitalization, Adherence

## Abstract

**Background:**

We conducted a prospective, randomized, controlled, two-group parallel trial investigating the effectiveness of a care management program employing cardiac care navigators providing post-discharge support to patients compared to standard care.

**Methods:**

The intervention commenced in 2019/2020 for 2862 patients hospitalized with heart failure, coronary heart disease, or cardiac arrhythmias in departments of cardiology across eight participating sites of a hospital group in Berlin, Germany. We analyzed the results using an intention-to-treat approach. The primary outcome was the all-cause rehospitalization rate after 12 months. Secondary outcomes included rehospitalizations due to one of the qualifying cardiac diagnoses, duration of rehospitalization, mortality, health-related quality of life, and several process indicators.

Trial data were collected from a combination of face-to-face and phone interviews conducted by hospital staff throughout the 12-month follow-up period using standardized questionnaires. Administrative claims data were provided by a large statutory health insurer. Outcomes for the intervention and control groups were compared using logistic regression, generalized linear models (GLMs) with a negative binomial distribution, ordinary least squares, and Cox proportional hazards regression.

**Results:**

Compared to the control group (*N* = 1294), the intervention group (*N* = 1256) had a lower rate of all-cause rehospitalizations (62.6% vs. 66.4%, *p* = 0.05) and shorter lengths of stay (14.49 vs. 16.89 days, *p* = 0.02) during the 12-month follow-up period. These differences were also present for rehospitalizations due to the cardiac diseases qualifying for study recruitment, with rehospitalization rates for the intervention and control groups being 58.0% vs. 61.4% (*p* = 0.08) and particularly pronounced for lengths of rehospitalization stay of 12.97 vs. 15.40 days (*p* = 0.01), respectively. Subgroup analyses indicated positive effects of the intervention for patients 70 years and older (*p* = 0.05), females (*p* = 0.06), and those with little or no German language proficiency (*p* = 0.03). Furthermore, we found positive effects on patients’ adherence to health-related behavioral recommendations (81.91% vs. 73.95%, *p* = 0.000).

**Conclusions:**

This study adds to the body of evidence indicating that care management interventions supporting patients as they transition from the inpatient to the outpatient sector can lower rehospitalizations, decrease length of rehospitalization stays, and improve adherence to post-discharge recommendations.

**Trial registration:**

German Clinical Trial Register, DRKS00020424. Registered 2020-06-18. (retrospectively registered).

**Supplementary Information:**

The online version contains supplementary material available at 10.1186/s12916-024-03691-7.

## Background

Cardiovascular diseases (CVDs) remain the leading cause of death worldwide and are the most frequent diagnoses in hospital settings [1]. Hospitalizations for CVDs, often signaling advanced disease stages, not only disrupt patients’ lives but also reduce their quality of life and generate high costs for healthcare systems [2–4]. As life expectancy increases, the prevalence and impact of CVDs are expected to grow. Therefore, effective management through timely diagnosis and treatment, the provision of and adherence to appropriate pharmacological treatment, and the facilitation of lifestyle modifications is essential to reduce the burden of these diseases.

Most patients with CVDs must navigate a complex network of healthcare providers, including general practitioners (GPs), cardiologists, and rehabilitation facilities. In fragmented healthcare systems, such as in Germany [5], cardiac patients often experience gaps in care, particularly after hospital discharge, resulting in increased rates of rehospitalization [6]. The period immediately following discharge is critical for integrating these patients into follow-up care, ensuring adherence to medical recommendations, and encouraging new and healthier lifestyle habits. However, this integration process is frequently obstructed not only by the fragmentation of healthcare systems but also by factors such as patients’ health status, the presence of comorbidities, language barriers, unfamiliarity with different healthcare services and how to access them, and limited educational background [7–9].

A relatively new approach to addressing this problem involves employing nurses or other professional medical staff as healthcare navigators to guide patients through the critical phases of their journey through the healthcare system. Such models of care have been introduced and piloted in various patient groups, including stroke survivors, the elderly, and individuals with rare diseases [10]. The reported outcomes of these programs include reductions in readmissions, increased quality of life, and positive healthcare experiences [10]. The effects of care management programs have also been investigated for specific populations of cardiac patients in various settings but have yielded mixed results. One study demonstrated that guiding heart failure patients through the healthcare system reduced hospitalizations and costs when compared to standard care [11]. In contrast, another study found an increase in physician visits following the implementation of a case management program for heart failure patients in Germany but no improvements in quality of life or self-care [12]. More recently, research by Stokes et al. (2016) showed that case management led to higher rates of non-elective hospitalizations and longer hospital stays among high-risk patients in one region in England [13]. To our knowledge, however, there appears to be scant evidence regarding the effectiveness of patient care management and case-management programs on the outcomes for cardiac patients following hospital discharge.

The “Cardiolotse” (CL; German for “cardiac care navigator”) program was developed to bridge gaps in the care received by cardiac patients, focusing specifically on the continuity and coordination of care following hospital discharge. The key component of the program was the CL, a specially trained medical care navigator whose primary task was to provide post-discharge support to patients with cardiac diseases as they transitioned from the inpatient to the outpatient care sector. This support consisted of guiding patients through the healthcare system, facilitating access to healthcare resources, offering prevention advice, and aiding in the self-management of their condition irrespective of educational level or social standing. The program was implemented as a randomized controlled trial (RCT) in a sample of cardiac patients in Berlin, Germany. In the present article, we report the results of this trial, which aimed to investigate the effectiveness of the CL program in comparison to usual care.

## Methods

### Study design, setting, and sample

The study was designed as a prospective, randomized, controlled, two-group parallel trial investigating the effects of a new care management program involving nurses or other medical staff who trained as cardiac care navigators and provided post-discharge support to cardiac inpatients.

**Fig. 1:**
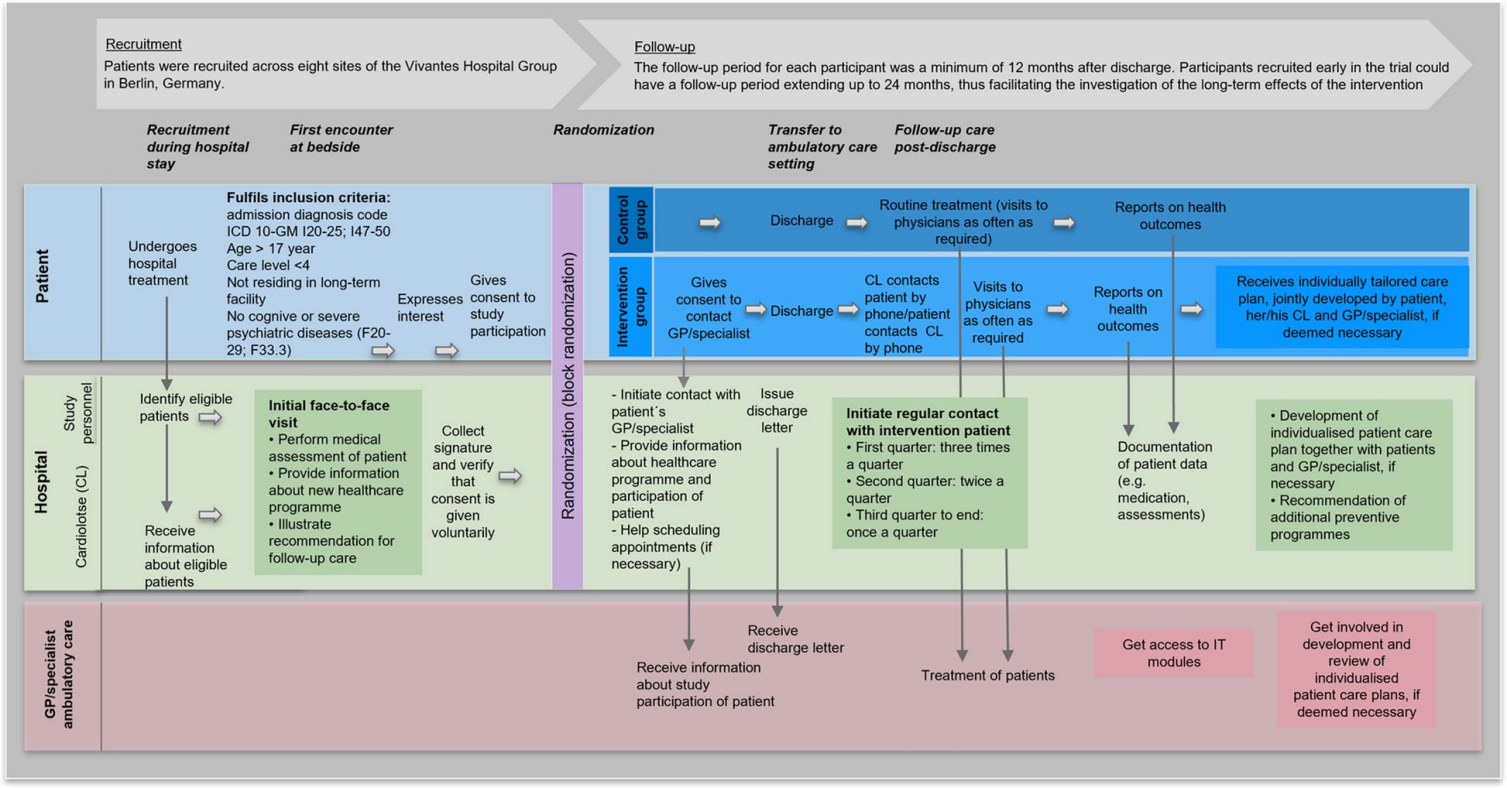
Description of the intervention

Patients were recruited across eight sites of the Vivantes Hospital Group in Berlin, Germany (see Fig. 1). Eligible patients were those aged 18 years or older who were hospitalized in the Departments of Cardiology at one of the participating sites for one or more of the relevant cardiac admission diagnoses (ICD-10-GM I20-I25, I47-I49) and insured by AOK Nordost. AOK Nordost is a statutory health insurer in Germany with a market share of approximately 24% [14]. Patients with severe psychiatric diseases, those in need of intensive nursing care, and residents of long-term care facilities were excluded.

Potentially eligible patients were identified by the CLs at each hospital site prior to discharge using the hospital information system. CLs visited potential participants to explain the study in detail and answer any questions related to enrolment, participation, and the process for study withdrawal. Eligible patients who provided written informed consent were then randomly allocated in a 1:1 ratio to either the intervention or control group. The Randoulette tool of the Institute for Medical Information Processing, LMU Munich, Germany, was used for simple block randomization with a block size of 10. The CLs received the allocation result of the patient immediately after entering the unique study identification number, the year of birth, and sex of the patient in the web-based tool. The specifics of the randomization algorithm were unknown to the CL. After assignment to either of the two arms, it was no longer feasible to blind patients or healthcare providers to the randomization status. Participants could withdraw from the study at any point during the intervention without providing justification.

### Intervention and control

Before the start of the intervention, the CLs underwent supplementary training that covered the following areas: communication techniques, understanding the physical and psychosocial health needs of people with CVDs, knowledge of preventive and rehabilitative treatment options, essential information about healthcare services and delivery mechanisms, and foundational principles in ethics and legal considerations. The time required for the training ranged from 160 to 240 h depending on existing qualifications. The staff eligible for this training were either nurses or medical or healthcare assistants. In order to be able to meet the individual needs of patients, great importance was attached to multilingualism when staffing the project.

The intervention began with an initial face-to-face meeting between the CL and the patient during the hospital stay. This meeting aimed to establish a personal connection to facilitate subsequent post-discharge communication. Intervention group patients received preventive advice tailored to their specific needs along with a booklet containing practical information, such as important telephone numbers. Information material such as booklets and leaflets were written with a specific focus on patient-oriented language and also translated in various languages. During the meeting, the CL also interviewed the patients, asking them questions regarding their vital parameters, adherence to health behavior recommendations, and satisfaction with care using a standardized questionnaire. Additionally, patients were provided with a letter to give to their GPs and cardiologists detailing the patients’ participation in the study and outlining how to cooperate with the program. The CL also had the option to involve the patient’s family members or relatives during the hospitalization to encourage active participation and integrate them into the planning for post-discharge care. The CLs were required to support the GPs’ or cardiologists’ advice to the patients, and the CLs were not entitled to alter these recommendations without prior contact with the prescribers.

After hospital discharge, the CL contacted intervention group patients by telephone at pre-defined intervals: monthly during the first quarter following discharge, every 6 weeks during the second quarter, and quarterly during the third and fourth quarters. Additionally, the CL was available through a dedicated hotline and scheduled office hours for any ad hoc medical inquiries.

During the regular telephone consultations, patients were asked standardized follow-up questions regarding their vital signs, medication adherence, and adherence to recommended health behaviors. The CL assisted patients in meeting their specific healthcare needs by facilitating access to health information and educational materials, motivating patients to overcome barriers and set goals for self-managing their condition, and advising on specialist referrals for any concerning symptoms. Additionally, the CL helped schedule appointments with GPs, cardiologists, or other healthcare providers.

Patients in the control group also had an initial face-to-face meeting with the CL, after which they received routine treatment, both pre- and post-hospital discharge. Treatment as usual consisted of all services and medication normally available in the ambulatory care setting in Germany. Participants in both groups could visit physicians as often as required.

Control group participants were also asked to complete follow-up questionnaires via telephone interviews at three months and 12 months post-discharge. These questionnaires were nearly identical to those administered to the intervention group.

The follow-up period for each participant was a minimum of 12 months after discharge. Participants recruited early in the trial could have a follow-up period extending up to 24 months, thus facilitating the investigation of the long-term effects of the intervention. Details of the trial design, intervention, and evaluation methods were described in a previously published study protocol [15] and the CONSORT checklist (Additional file 1).

### Outcomes

The primary outcome of the study was the 12-month all-cause rehospitalization rate. The study aimed to achieve a 20% relative reduction (initially estimated at 25%) in this rate. To detect this reduction with a significance level of 5% and power of 80%, a minimum of 1094 patients were required for the analysis. It was anticipated that approximately 2% of the patients would be non-adherent due to the inability of the CL to offer support. Furthermore, a 5% contamination of the control group by other healthcare interventions in the Berlin area was assumed. To adjust for these factors, the target sample size was multiplied by a factor of 1.16. Accounting for an expected loss to follow-up of 13%, a sample size of 2908 was required, as determined by our sample size calculation [15].

Using administrative claims data, we examined the all-cause rehospitalization rate at intervals of 30 days, 3 months, 6 months, 12 months, and, for the subgroup of patients with an observation period extending to 24 months, at 24 months post-discharge. Additionally, we assessed the rate of rehospitalizations related to the cardiac diagnoses qualifying for inclusion. We also investigated mortality and the duration of rehospitalization stays. We examined health-related quality of life using the German telephone version of the EuroQol questionnaire (EQ-5D-5L) [16].

Furthermore, we assessed process indicators along the patient pathway using the trial data collected in this study. This assessment included whether patients experienced changes in medication after discharge, their adherence to recommendations for prevention measures and smoking cessation, and the number of visits to GPs and specialists. We also evaluated physician visits for the full sample using routine data. Additionally, we compared the continuity of care between the groups using the Continuity of Care Index and the Usual Provider Index [17]. The Continuity of Care Index measures the dispersion of visits across different providers, calculated by dividing the number of visits to each physician by the total number of visits a patient had overall. The index ranges from just above 0, indicating visits to a variety of doctors, to 1, indicating all visits were made to the same physician. The Usual Provider Index measures continuity of care with one usual provider. It divides the number of visits to the usual provider by the total number of visits to all providers. This index also ranges from 0 (all visits made to different providers) to 1 (all visits made to the usual provider). The usual provider in this study was defined as the physician with the maximum number of visits during the observation period. Our analyses of these indices were restricted to visits to GPs, internists, and cardiologists.

Subgroup analyses were conducted across the various diagnosis groups and subgroups, which we defined based on patient characteristics such as age, gender, care levels, socioeconomic status, and proficiency in German. Furthermore, we defined subgroups based on whether they actively participated in the intervention or were unavailable for the intervention due to prolonged hospitalizations (i.e., exceeding 28 days during the observation period).

Recruitment took place between January 2019 and March 2020. The final recruitment phase and follow-up period coincided with the COVID-19 pandemic. Due to the potential impact of the pandemic on the selection of patients hospitalized during this period and subsequent variations in rehospitalization rates, we additionally investigated the subgroup of patients recruited in the first quarter of 2019 because their follow-up data were not affected by the pandemic.

The study also included a process evaluation and an economic evaluation, the results of which are reported elsewhere.

### Data collection

Trial data were collected in the face-to-face and telephone interviews during the follow-up period. Additionally, AOK Nordost provided claims data covering patient information, hospital records, prescriptions, diagnoses, and ambulatory care details. The trial data and administrative claims data were merged at an independent trust center and analyzed pseudonymously. Data from individuals who withdrew from the study were excluded from the evaluation.

### Confounding factors

Data such as socioeconomic status, including levels of education and employment status, were collected at baseline as potential confounding factors. Additionally, gender, age, and morbidity were identified using routine claims data. To control for comorbidity, we employed the Charlson Comorbidity Index, using ambulatory and hospital diagnoses from the year prior to enrolment in the study to calculate comorbidity scores [18]. We used the Elixhauser Comorbidity Index as an alternative comorbidity measure.

### Statistical analysis

We assessed differences in patient characteristics, the incidence of COVID-19, and the included cardiac diseases at baseline using *t*-tests.

Our analysis of the primary and secondary outcomes was conducted following an intention-to-treat approach. We examined differences in rehospitalization rates (a binary outcome) using logistic regression models adjusting for age, sex, and the respective comorbidity score. In our sensitivity analyses, we compared the results of our main models with those unadjusted for these variables and, as an alternative to the Charlson index, those obtained using the Elixhauser Comorbidity Score (Table A1 in Additional file 2).

Additionally, we analyzed the number of rehospitalizations (a count variable) during the follow-up period using generalized linear models (GLMs) with a negative binomial distribution to account for overdispersion. Secondary outcomes were evaluated using standardized regression models. To test for differences in the time until death between the groups, we conducted Cox proportional hazards analyses.

Health-related quality of life was analyzed using ordinary least squares regression, with missing quality-of-life values imputed via a multiple imputation model following Faria et al. [19]. Additionally, we applied alternative imputation techniques, the results of which are reported in the Additional file 3 Table A2.

Process indicators, derived from trial data and routine data observations, included measures such as physician visits and continuity of care indices. These were analyzed using ordinary least squares (OLS) regression for continuous outcomes and logistic regression for binary outcomes.

All analyses were conducted using Stata 14.

## Results

### Trial population and baseline characteristics

**Fig. 2:**
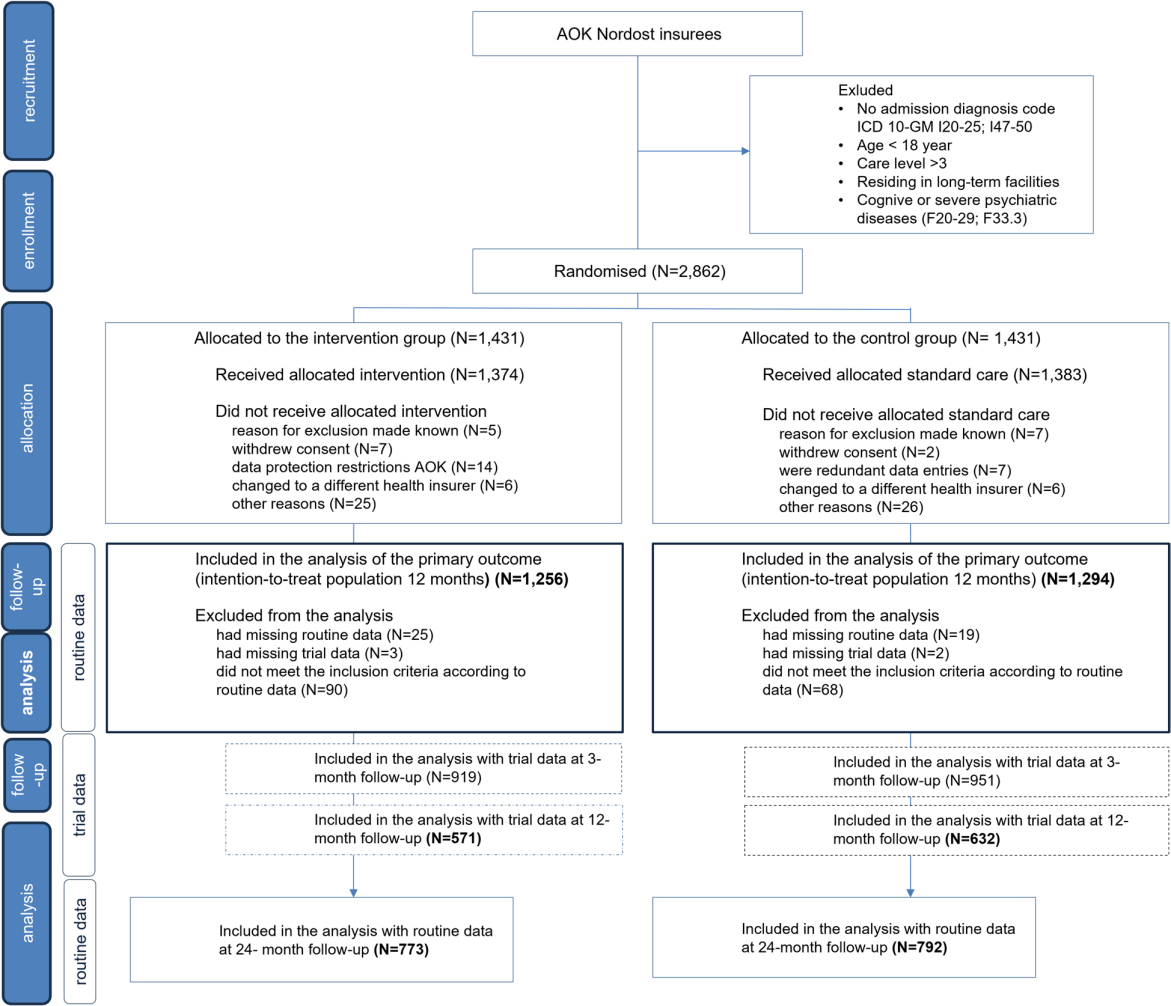
Diagram of patient flow through the trial

A total of 2862 patients were identified as eligible for the study and were subsequently randomly allocated to either the intervention or control group. Due to various factors such as withdrawal of consent by the patient, changes of insurer during the follow-up period, or data protection limitations, 10% of the randomized patients were excluded from the evaluation. As a result, 1256 patients remained in the intervention group and 1294 in the control group (study population for the primary and secondary outcomes). Additionally, trial data were available for 81% of the surviving patients in both groups at the 3-month follow-up and for 61% of the surviving patients in the intervention group and 65% in the control group at the 12-month follow-up. The flow of patients through the study is depicted in Fig. 2.

The mean (SD) age of the study population (*N* = 2550) was 73.5 (12.66) years, 54% of whom were men. In total, 46% of the patients had heart failure, 65% coronary heart disease, and 54% cardiac arrhythmias. Almost 73% of the patients were retired. At the time of assignment to the study, 46% of the study population did not have a designated level of need for long-term care. The majority of patients (65.8%) had no schooling or only basic secondary education (Volks-, Hauptschule) as their highest level of school education. The remaining 35% had a higher level of school education, such as intermediate secondary education (Mittlere Reife, Realschule), vocational/technical secondary education (Fachoberschule), or secondary education qualifying for university admission (Abitur/EOS). With regard to professional qualifications, 90.2% of the patients had either no qualification or only an apprenticeship as their highest qualification. The remaining 9.8% of patients had a higher professional qualification, such as a degree from a technical school (Fachschule), technical college/university of applied sciences (Fachhochschule), or university (Universität). The CL rated the German language proficiency of almost 92% of the study participants as good or very good, whereas the remaining participants had mediocre, hardly any, or no proficiency.

The study participants had a high burden of coexisting diseases, with a mean Charlson index of 3.31 and Elixhauser index of 5.45.

The main demographic and clinical characteristics of the patients at baseline are shown in Table 1. There were no significant differences in the distribution of sociodemographic or clinical characteristics between the intervention and control groups.

**Table 1:** Demographic and clinical characteristics of the study population at baseline

	Intervention group (*N*=1,256)	Control group (*N*=1,294)	Difference in means
Socio-demographic characteristics			
Age in years^a^	73.48 (12.90)	73.54 (12.42)	-0.00 (0.001)
Female sex, n (%)^a^	592 (47.1)	571 (44.1)	0.03 (0.020)
Retired, n(%)^a^	902 (71.8)	954 (73.7)	-0.019 (0.018)
Level of need for long-term care, n (%)^a^			
Level 1 (minor impairment of independence or abilities)	151 (12.0)	158 (12.2)	-0.012 (0.032)
Level 2	363 (28.9)	384 (29.7)	-0.014 (0.023)
Level 3	121 (9.6)	128 (9.9)	-0.014 (0.035)
Level 4	26 (2.1)	32 (2.5)	-0.052 (0.067)
Level 5 (severe impairments associated with special requirements for nursing care)	5 (0.4)	3 (0.2)	0.125 (0.178)
Highest level of school education, n(%)^b^			
*No or only basic schooling:* No school leaving certificate or only basic secondary education (Volks-, Hauptschule)	673 (64.2)	696 (67.4)	-0.002 (0.020)
*Higher level of school education*: Intermediate secondary education (Mittlere Reife, Realschule), vocational/technical secondary education (Fachoberschule), secondary education qualifying for university admission (Abitur/EOS)	376 (35.8)	336 (32.6)	0.002 (0.020)
Highest professional qualification, n (%)^b^			
*No or only basic professional qualification:* No training or only an apprenticeship (Lehre)	943 (90.0)	927 (90.4)	0.044 (0.020)
*Higher professional qualification:* Vocational school (Berufsfachschule), Technical school (Fachschule), technical college/university of applied sciences (Fachhochschule), university (Universität)	105 (10.0)	99 (9.6)	-0.044 (0.022)
German language proficiency^b^	1.321 (0.785)	1.283 (0.731)	0.038 (0.032)
Medical characteristics			
Length of hospital stay (index hospitalization)^a^ in days	7.975 (9.181)	8.056 (8.879)	-0.082 (0.338)
Charlson Comorbidity Index^a^	3.232 (2.690)	3.380 (2.804)	-0.148 (0.109)
Elixhauser Comorbidity Index^a^	5.424 (3.159)	5.473 (3.109)	-0.050 (0.124)
Patient is a smoker, n (%)^a^	271 (21.6)	268 (20.7)	0.015 (0.017)
Patients with heart failure (ICD-10 I50), n (%)^a^	583 (46.4)	588 (45.4)	0.010 (0.020)
Patients with coronary heart disease (ICD-10 I20-I25), n (%)^a^	818 (65.1)	847 (65.5)	-0.003 (0.019)
Patients with cardiac arrhythmias (ICD-10 I47-I49), n (%)^a^	689 (54.9)	690 (53.3)	0.015 (0.020)
Patients with a COVID-19 diagnosis during 12 months follow-up, n (%)^a^	207 (16.5)	184 (14.2)	0.028 (0.014)

Categorical variables with label n (%), continuous variables mean (standard deviations/errors in parentheses); German language proficiency assessed by the CL: 1 very good, 2 good, 3 average, 4 little, 5 none. Disease groups: Patients may have had multiple diagnoses during the initial stay

^a^Routine claims data from AOK Nordost

^b^Trial data

### Follow-up and trial outcomes

Each CL conducted an average of 10 telephone consultations with patients in the intervention group surviving during the follow-up period. Within the 12 months, 26% of the intervention patients were identified as inactive, indicating that they could not be contacted or were unreachable for a certain period. Furthermore, 11.2% of the patients were hospitalized for 28 or more days during the observation period and thus classified as patients with especially long hospital stays.

On average, a smaller proportion of the intervention group experienced rehospitalization in the year after enrollment in the program compared to the control group (63% versus 66%). However, this difference in the primary outcome was not consistently significant across the models: It was significant in the unadjusted analyses (*p* = 0.045) but not in the adjusted analyses (*p* = 0.067, *p* = 0.056). More than 40% of the patients in the intervention and control groups experienced a rehospitalization by 3 months. After 24 months, more than two thirds of the study population had experienced a rehospitalization. Furthermore, the average number of rehospitalizations in the intervention group was 1.44 compared to 1.55 in the control group during 12 months of follow-up. The results for the primary and secondary outcomes are reported in Table 2.

**Table 2:** Primary and secondary outcomes of the trial

	Intervention group	Control group	Effect size (95% CI)	Adjusted effect size (95% CI)
**Primary outcome**				
** Rehospitalization rate (all causes)**^**αα**^				
** 12 months**	**0.626 (0.484)**	**0.664 (0.473)**	0.847** (0.720–0.996)	0.857* (0.726–1.011)
30 days	0.243 (0.429)	0.248 (0.423)	0.972 (0.812–1.164)	0.980 (0.816–1.177)
3 months	0.439 (0.497)	0.460 (0.499)	0.921 (0.788–1.077)	0.934 (0.797–1.094)
6 months	0.524 (0.500)	0.555 (0.497)	0.883 (0.755–1.032)	0.895 (0.763–1.049)
24 months (*N* = 1565)	0.728 (0.445)	0.735 (0.442)	0.967 (0.773–1.210)	0.983 (0.781–1.237)
**Secondary outcomes**				
** Number of rehospitalizations (all causes)**^**ββ**^				
** 12 months**	**1.439 (1.731)**	**1.553 (1.866)**	**0.927 (0.844–1.017)**	**0.943 (0.861–1.034)**
30 days	0.272 (0.507)	0.279 (0.523)	0.976 (0.844–1.128)	0.986 (0.854–1.139)
3 months	0.632 (0.859)	0.647 (0.861)	0.977 (0.880–1.085)	0.997 (0.899–1.106)
6 months	0.964 (1.233)	1.002 (1.252)	0.963 (0.873–1.062)	0.986 (0.894–1.086)
24 months (*N* = 1565)	2.223 (2.526)	2.177 (2.469)	1.021 (0.912–1.143)	1.027 (0.921–1.146)
**Time (in days)** **to rehospitalization** **(all causes)**^γγ^				
** 12 months**	**85.850 (90.078)**	**88.943 (93.552)**	**0.915 (0.831–1.008)**	**0.925 (0.840–1.019)**
30 days	13.176 (8.310)	13.466 (8.181)	0.986 (0.846–1.149)	0.996 (0.855–1.160)
3 months	34.679 (24.359)	35.307 (23.775)	0.944 (0.866–1.030)	0.954 (0.876–1.040)
6 months	50.238 (43.305)	53.001 (45.001)	0.923** (0.861–0.989)	0.933* (0.872–1.000)
24 months (*N* = 1565)	164.510 (186.557)	160.365 (181.336)	0.978 (0.886–1.078)	0.989 (0.898–1.090)
** Combined outcome: rehospitalization rate all causes or death**^**αα**^				
** 12 months**	**0.662 (0.473)**	**0.694 (0.461)**	0.862* (0.730–1.018)	**0.875 (0.738–1.038)**
30 days	0.264 (0.441)	0.267 (0.443)	0.980 (0.822–1.169)	0.990 (0.828–1.184)
3 months	0.469 (0.499)	0.485 (0.500)	0.936 (0.802–1.094)	0.953 (0.813–1.116)
6 months	0.557 (0.497)	0.583 (0.493)	0.902 0.771–1.055)	0.918 (0.782–1.078)
24 months (*N* = 1565)	0.728 (0.445)	0.735 (0.442)	0.967 (0.773–1.210)	0.983 (0.781–1.237)
** Rehospitalization rate for hospitalizations for included cardiac diagnoses**^**αα**^				
** 12 months**	**0.580 (0.494)**	**0.614 (0.487)**	0.868* (0.741–1.017)	**0.879 (0.747–1.033)**
30 days	0.219 (0.414)	0.223 (0.417)	0.975 (0.808–1.175)	0.982 (0.813–1.188)
3 months	0.406 (0.491)	0.421 (0.494)	0.940 (0.802–1.100)	0.952 (0.811–1.117)
6 months	0.488 (0.500)	0.515 (0.500)	0.899 (0.770–1.050)	0.911 (0.778–1.068)
24 months (*N* = 1565)	0.665 (0.472)	0.684 (0.465)	0.915 (0.741–1.131)	0.932 (0.749–1.161)
** Number of rehospitalizations for included cardiac diagnoses**^**ββ**^				
** 12 months**	**1.230 (1.541)**	**1.374 (1.724)**	0.895** (0.812–0.987)	0.908** (0.825–0.999)
30 days	0.244 (0.484)	0.249 (0.491)	0.979 (0.840–1.142)	0.984 (0.844–1.146)
3 months	0.561 (0.798)	0.583 (0.823)	0.961 (0.861–1.072)	0.979 (0.878–1.092)
6 months	0.844 (1.116)	0.901 (1.187)	0.937 (0.845–1.038)	0.957 (0.865–1.059)
24 months (*N* = 1565)	1.873 (2.336)	1.896 (2.268)	0.990 (0.877–1.117)	0.995 (0.884–1.119)
** Mortality (any cause)**^**εε**^				
** 12 months**	**0.151 (0.358)**	**0.157 (0.364)**	**0.914 (0.787–1.064)**	**0.939 (0.807–1.092)**
30 days	0.038 (0.192)	0.042 (0.200)	0.914 (0.620–1.349)	0.937 (0.635–1.382)
3 months	0.072 (0.258)	0.072 (0.258)	0.996 (0.745–1.331)	1.018 (0.762–1.361)
6 months	0.098 (0.297)	0.099 (0.299)	0.986 (0.770–1.243)	1.013 (0.791–1.297)
24 months (*N* = 1565)	0.275 (0.446)	0.285 (0.452)	0.957 (0.816–1.123)	0.990 (0.844–1.163)
** Number of days of rehospitalization (LOS) for any cause (all causes)**^δδ^				
** 12 months**	**14.49 (24.32)**	**16.89 (28.98)**	0.858** (0.752–0.978)	0.859**(0.750–0.985)
30 days	3.301(8.138)	3.873 (10.03)	0.852 (0.700–1.037)	0.861 (0.746–1.046)
6 months	9.932 (18.65)	11.58 (22.23)	0.857** (0.740–0.994)	0.863* (0.742–1.005)
24 months (*N* = 1565)	19.11 (31.20)	20.31 (34.85)	0.941 (0.797–1.111)	0.940 (0.798–1.107)
** Number of days of rehospitalization (LOS) for included cardiac diagnoses**^δδ^				
** 12 months**	**12.97 (22.56)**	**15.40 (27.43)**	0.843** (0.735–0.966)	0.836** (0.725–0.964)
30 days	3.053 (7.804)	3.638 (9.921)	0.839* (0.684–1.030)	0.840 (0.675–1.044)
6 months	8.984 (17.24)	10.59 (20.88)	0.849** (0.730–0.987)	0.849** (0.724–0.994)
24 months (*N* = 1565)	16.81 (29.35)	18.26 (32.69)	0.921 (0.773–1.097)	0.914 (0.769–1.086)
** Health-related quality of life (EQ 5D-5L)**^**a** δδ^				
at baseline	0.747 (0.008)	0.750 (0.009)	− 0.003 (− 0.028-0.022)	− 0.003 (− 0.028-0.021)
at 3 months	0.640 (0.011)	0.626 (0.011)	0.013 (− 0.017-0.043)	0.010 (− 0.018-0.039)
at 12 months	0.600 (0.013)	0.564 (0.012)	0.036* (− 0.000-0.072)	0.032* (− 0.003-0.066)
** Visits to GPs and specialists 12 months after discharge**				
Patients with a GP visit^αα^	0.951(0.215)	0.962 (0.191)	0.771 (0.525–1.132)	0.748 (0.509–1.100)
Patients with a specialist visit^αα^	0.448 (0.498)	0.449 (0.498)	0.997 (0.853–1.165)	0.996 (0.850–1.167)
Number of GP visits^ββ^	14.60 (9.484)	14.54 (10.33)	1.004 (0.952–1.058)	1.005 (0.954–1.058)
Number of specialist visits^ββ^	1.163 (1.756)	1.175 (1.912)	0.990 (0.876–1.118)	0.993 (0.880–1.121)
Continuity of care index (GPs)^δδ^ (*N* = 2406)	0.683 (0.251)	0.686 (0.252)	− 0.003 (− 0.023-0.017)	− 0.004 (− 0.024-0.016)
Continuity of care index (GPs, cardiologists)^δδ^ (*N* = 2396)	0.771 (0.251)	0.770 (0.253)	0.000 (− 0.020-0.021)	− 0.001 (− 0.021-0.019)
Usual provider index (GPs) (*N* = 2406)^δδ^	0.771 (0.208)	0.774 (0.209)	− 0.003 (− 0.020-0.013)	− 0.004 (− 0.020-0.013)
Usual provider index (cardio.)^δδ^ (*N* = 2405)	0.833 (0.204)	0.833 (0.207)	0.000 (− 0.016-0.017)	− 0.001 (− 0.017-0.016)
** Adherence to medical advice**^**a** αα^				
Medication unchanged after discharge (*N* = 2101)	0.852 (0.355)	0.795 (0.404)	1.480*** (1.180–1.856)	1.489*** (1.186–1.870)
Adherence to prevention recommendations (*N* = 1994)	0.818 (0.386)	0.738 (0.440)	1.661*** (1.338–2.062)	1.647*** (1.326–2.046)
Smoking cessation (*N* = 1079, excluding non-smoker)	0.307 (0.462)	0.306 (0.461)	0.957 (0.738–1.241)	0.913 (0.694–1.201)
Visited GP as recommended (*N* = 1994)	0.946 (0.227)	0.950 (0.218)	0.829 (0.563–1.221)	0.812 (0.553–1.194)
Visited a specialist as recommended (*N* = 1995)	0.783 (0.412)	0.711 (0.453)	1.465*** (1.195–1.797)	1.471*** (1.199–1.806)

Values for intervention and control group indicate the mean values. Standard deviations/errors in parentheses

Adjusted: Effect sizes are adjusted for age, gender and morbidity (Charlson comorbidity index)

^***^p < 0.01, **p < 0.05, *p < 0.1

Model: ^αα^Logistic regression, reported coefficients presented as odds ratios; ^ββ^GLM, negative binomial distribution with log link, reported coefficients presented as incidence ratios; ^γγ^Cox proportional hazards model, reported coefficients presented as hazard ratios; ^δδ^OLS model, robust; ^εε^Exponential survival model, reported coefficients presented as hazard ratios (Kaplan-Meier survival curves Figures A1 and A2 in the additional files 4 and 5)

Note: ^a^Trial data. Adherence to medical advice: assessed in phone call after 12 months (9–21 months in cases where 12-month call was not conducted) in the intervention group, control group after 12 months

In the trial population, 393 patients died during the 12-month follow-up period (190 in the intervention, 203 in the control group). Information on the causes of death was not available to the study team. However, we found no significant difference in mortality between the intervention and control groups at any time point, also when considering the disease subgroups or when analyzing time until death.

After 12 months, a significantly lower number of rehospitalizations related to the included cardiac diagnoses was found in the intervention compared to the control group. Additionally, the average length of rehospitalization stay was significantly shorter in the intervention group (14.49 days) than in the control group (16.89 days).

Health-related quality of life decreased in both groups over the year. However, after 1 year, patients in the intervention group reported on average a higher health-related quality of life compared to the control group (0.036, *p* = 0.051) also with different imputation methods applied. This difference exceeds the values considered to represent a minimum clinically important difference (0.03–0.54) [20, 21].

The process indicators based on claims data showed only small differences between the intervention and control groups. None of the outcomes related to post-discharge ambulatory care during the follow-up period (i.e., the proportion of patients who visited a GP/a cardiologist, the number of visits to the relevant physicians, and continuity of care) differed significantly between the two groups.

However, the analysis of trial data showed significant differences in physician visits: 78% of the intervention group compared to 71% of the control group reported having visited a cardiologist during the follow-up period. Moreover, a significantly higher percentage of patients in the intervention group (81.91%) reported having adhered to prevention recommendations compared to the control group (73.95%) (*p* = 0.000).

### Results of the subgroup analyses

**Fig. 3:**
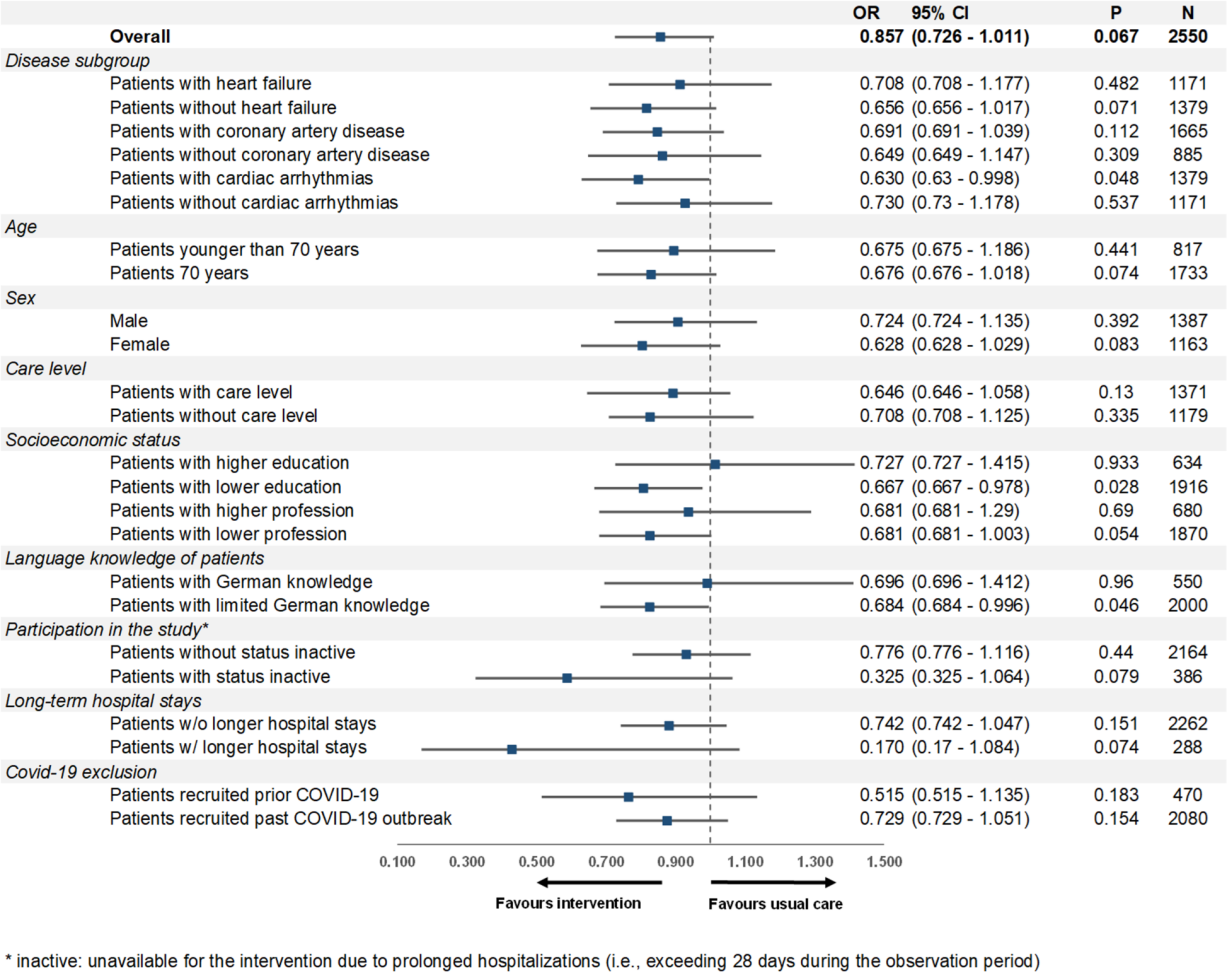
Subgroup analysis of rehospitalization rates, univariate results

Figure 3 presents the results of the univariate subgroup analyses. Among the cardiac disease subgroups, patients with heart failure experienced the highest rehospitalization rates. Patients with cardiac arrhythmias in the intervention group had significantly lower rehospitalization rates than those in the control group (*p* = 0.032). We also found lower rehospitalization rates in females (*p* = 0.061) and older patients (*p* = 0.048) in the intervention group compared to their counterparts in the control group.

Patients with no schooling or only basic schooling, as well as patients who had no professional qualification or only a basic professional qualification, showed higher rehospitalization rates compared to those with higher levels of schooling or higher professional qualifications, respectively.

When designing the study, it was assumed that patients with limited proficiency in German would particularly benefit from the healthcare program. Indeed, subgroup analyses show that intervention patients with little or no German language proficiency had lower rehospitalization rates than those in the control group (*p* = 0.030).

Lastly, intervention patients recruited before or after the onset of the COVID-19 pandemic experienced lower rehospitalization rates on average compared to the control group, regardless of the time of recruitment.

## Discussion

CVDs were responsible for an estimated 32% of deaths globally in 2019 [1] and are associated with high rates of hospital readmission [22, 23]. In Germany’s fragmented healthcare system, an important concern is whether inadequate care coordination and information exchange during the transition from inpatient to outpatient care are contributing to these high rehospitalization rates, resulting in suboptimal treatment outcomes. The “Cardiolotse” (cardiac care navigator) program was introduced to improve this transition by facilitating access to healthcare resources and the exchange of information, supplementing existing discharge services and support in the subsequent period. To assess the effectiveness of the “Cardiolotse” program in addressing these challenges, we conducted an RCT to evaluate its impact on reducing rehospitalizations and improving treatment coordination and patient outcomes in the 12-month post-discharge period.

In our study, the rate of rehospitalization in the intervention group was three percentage points lower in the year after enrollment in the program compared to the control group. Moreover, the average length of rehospitalization stay in the intervention group was 2.4 days shorter compared to the control group. The difference was especially pronounced in patients with any of the included cardiac diseases (i.e., heart failure, coronary heart disease, and cardiac arrhythmias). Additionally, we found improvements in adherence to post-discharge behavioral recommendations in the intervention group. Importantly, we found that the positive effect of the intervention with regard to the rate of rehospitalization could be detected in all patient subgroups, including those with cardiac arrhythmias, older and female patients, and those with little or no proficiency in German. Subgroup analyses showed that patients who experienced hospital stays longer than 28 days also appeared to benefit from the intervention.

Unlike the findings from comparable case management programs, such as those reported by Hendricks et al., our study did not identify significant differences in overall rehospitalization or mortality rates [11]. However, in contrast to the rehospitalization rates of 6% to 16% reported in the Hendricks et al. study, the rates in our trial exceeded 60% probably due to the higher age and more comorbidities of our patient cohort. It is conceivable that this high likelihood of rehospitalization may have limited the potential for the CL program to mitigate such outcomes.

The intervention took place between January 2019 and March 2021, coinciding with the COVID-19 pandemic and leading to widely observed changes in patient behavior, adherence to health recommendations, and patterns of hospitalization and mortality rates [24–26]. These pandemic-related changes may also have limited the potential for the CL program to reduce rehospitalizations.

For example, the impact of the pandemic on patient outcomes in our study warrants consideration. Although we observed no differences in comorbidity levels before and after the onset of the pandemic, the heightened risk associated with cardiac diseases and older age for severe COVID-19 outcomes suggests that rehospitalizations during the pandemic likely involved more severe, unavoidable cases, as elective and less urgent cases were presumably deferred. To assess the effectiveness of the CL program in the absence of the influence of the pandemic, we conducted subgroup analyses including only those patients who were recruited in the first quarter of 2019 and were thus observed before the onset of the pandemic.

Furthermore, the pandemic might have influenced rehospitalization rates either by leading to the avoidance and postponement of hospitalizations whenever possible or by causing an increase in hospitalizations due to COVID-19 infections. However, our analysis indicates that COVID-19 infections were distributed evenly between the intervention and control groups, with only a small fraction of participants (*n* = 174) hospitalized due to COVID-19.

Furthermore, the impact of the pandemic may have extended to the secondary outcomes of our study. As observed by Muschol and Gissel (2021) in outpatient specialist care across Germany [27], the pandemic likely contributed to increased waiting times for appointments and a reduction in the number of ambulatory care visits. In our study, no differences were detected in the continuity of care as measured through care indices between the intervention and control groups, a result that could reflect the exceptional circumstances of the pandemic. Nonetheless, we found slightly higher rates of cardiologist visits among patients in the intervention group during the follow-up period.

The pandemic may have also influenced patients’ adherence to recommended changes in lifestyle behavior. On the one hand, the increased risk of SARS-CoV-2 infection may have motivated patients to alter their behavior, particularly related to smoking status, obesity, and cardiovascular disease, independently of their allocation to the study groups, especially during the period before vaccines became available. This situation coincided with the majority of the patients’ follow-up periods. On the other hand, pandemic-induced closures and restrictions may have hampered efforts to adopt healthier lifestyle habits. For example, sports courses were suspended for extended periods during the follow-up period of most patients.

This study is subject to several further limitations. First, its implementation in one hospital group with patients from one statutory health insurer and exclusively in Berlin limits generalizability of the findings. The demographic and socioeconomic profile of these patients may differ from that of the general public in terms of age, morbidity, and socioeconomic characteristics. To address this, we conducted several subgroup analyses focusing on these characteristics. Second, while we conducted various analyses to gain further insights into the mechanisms underpinning the effect of the “Cardiolotse” program, the study was powered to detect differences in the primary study outcome only, namely the all-cause rehospitalization rate after one year. Additionally, it is possible that the full effects of the “Cardiolotse” program may require more than one year to be detected, a hypothesis supported by observed changes in reported behavior that could potentially reduce rehospitalizations in the longer term. Nonetheless, these findings should be interpreted with caution due to the potential for selection bias and the likelihood of response bias in reported outcomes.

## Conclusions

Despite the challenges posed by the COVID-19 pandemic, the “Cardiolotse” program significantly reduced rehospitalization rates for patients with cardiac diseases. Moreover, we observed significant effects of the intervention for older patients, females, patients with little or no language proficiency in German, and those with especially long hospital stays compared to their respective counterparts who received standard care. These findings highlight the effectiveness of the program in supporting vulnerable patient groups. Furthermore, our secondary outcomes suggest that the program led to behavioral changes that may have an additional long-term impact on rehospitalization rates, even under challenging conditions like those in the COVID-19 pandemic. The results of this study may be used to tailor care management programs to future patient groups. Particularly older patients with cardiac diseases and patients facing language barriers may benefit from this type of intervention.

## Supplementary Information

Additional file 1. CONSORT Checklist for the study

Additional file 2: Table A1. Regression results with different adjustment for different comorbidity scores

Additional file 3: Table A2. Imputation values of the health-related quality of life (EQ-5D-5L)

Additional file 4: Figure A1. Kaplan-Meier Survival curves

Additional file 5: Figure A2. Time to Rehospitalization

## Data Availability

The data that support the findings of this study are available from the statutory health insurance AOK Nordost and the Vivantes hospital group and are not publicly available. Restrictions apply to the availability of these data, which were used under license for this study. Data are however available from the authors upon reasonable request and with permission of the AOK Nordost, Vivantes hospital group and its supervisory authority.

## Abbreviations

CL: “Cardiolotse” (German for “cardiac care navigator”)
CVDs: Cardiovascular diseases
CI: Confidence interval
GPs: General practitioners
GLMs: Generalized linear models
OLS: Ordinary least squares
RCT: Randomized controlled trial
SD: Standard deviations
