# Supplementary material for: Reducing rehospitalization in cardiac patients: a randomized, controlled trial of a cardiac care management program (“Cardiolotse”) in Germany
Source: BMC Med. 2024 Oct 21;22:480. doi: 10.1186/s12916-024-03691-7 (PMC11492482; doi:10.1186/s12916-024-03691-7)
Supplement: Supplementary file 2 — Additional file 2: Table A1. Regression results with different adjustment for different comorbidity scores [file 12916_2024_3691_MOESM2_ESM.docx]

**Additional file 2**

Table A1: Regression results with different adjustment for different comorbidity scores

|  |  | Intervention group | Control group | Adjusted effect size (95% CI) (Age, gender, Charlson index) | Adjusted effect size (95% CI) (Age, gender, Elixhauser index) |
| --- | --- | --- | --- | --- | --- |
| **Primary outcome** | | | | |  |
| **Rehospitalization rate (all causes)^αα^** | | | | |  |
|  | **12 months** | **0.626 (0.484)** | **0.664 (0.473)** | **0.857* (0.726-1.011)** | **0.850* (0.720-1.004)** |
|  | 30 days | 0.243 (0.429) | 0.248 (0.423) | 0.980 (0.816-1.177) | 0.971 (0.809-1.166) |
|  | 3 months | 0.439 (0.497) | 0.460 (0.499) | 0.934 (0.797-1.094) | 0.927 (0.791-1.086) |
|  | 6 months | 0.524 (0.500) | 0.555 (0.497) | 0.895 (0.763-1.049) | 0.887 (0.757-1.040) |
|  | 24 months (N=1,565) | 0.728 (0.445) | 0.735 (0.442) | 0.983 (0.781-1.237) | 0.980 (0.778-1.236) |
| **Secondary outcomes** | | | | |  |
| **Number of rehospitalizations (all causes)^ββ^** | | | | |  |
|  | **12 months** | **1.439 (1.731)** | **1.553 (1.866)** | **0.943 (0.861-1.034)** | **0.939 (0.857-1.028)** |
|  | 30 days | 0.272 (0.507) | 0.279 (0.523) | 0.986 (0.854-1.139) | 0.978 (0.846-1.130) |
|  | 3 months | 0.632 (0.859) | 0.647 (0.861) | 0.997 (0.899-1.106) | 0.991 (0.894-1.100) |
|  | 6 months | 0.964 (1.233) | 1.002 (1.252) | 0.986 (0.894-1.086) | 0.979 (0.889-1.078) |
|  | 24 months (N=1,565) | 2.223 (2.526) | 2.177 (2.469) | 1.027 (0.921-1.146) | 1.018 (0.913-1.135) |
| **Combined outcome: Rehospitalization rate all causes and death^αα^** | | | | |  |
|  | **12 months** | **0.662 (0.473)** | **0.694 (0.461)** | **0.875 (0.738-1.038)** | **0.850* (0.720-1.004)** |
|  | 30 days | 0.264 (0.441) | 0.267 (0.443) | 0.990 (0.828-1.184) | 0.971 (0.809-1.166) |
|  | 3 months | 0.469 (0.499) | 0.485 (0.500) | 0.953 (0.813-1.116) | 0.927 (0.791-1.085) |
|  | 6 months | 0.557 (0.497) | 0.583 (0.493) | 0.918 (0.782-1.078) | 0.888 (0.757-1.040) |
|  | 24 months (N=1,565) | 0.728 (0.445) | 0.735 (0.442) | 0.983 (0.781-1.237) | 0.980 (0.777-1.236) |
| **Rehospitalization rate for index diagnosis^αα^** | | | | |  |
|  | **12 months** | **0.580 (0.494)** | **0.614 (0.487)** | **0.879 (0.747-1.033)** | **0.872 (0.741-1.027)** |
|  | 30 days | 0.219 (0.414) | 0.223 (0.417) | 0.982 (0.813-1.188) | 0.974 (0.806-1.177) |
|  | 3 months | 0.406 (0.491) | 0.421 (0.494) | 0.952 (0.811-1.117) | 0.945 (0.805-1.109) |
|  | 6 months | 0.488 (0.500) | 0.515 (0.500) | 0.911 (0.778-1.068) | 0.904 (0.771-1.059) |
|  | 24 months (N=1,565) | 0.665 (0.472) | 0.684 (0.465) | 0.932 (0.749-1.161) | 0.928 (0.744-1.159) |
| **Number of rehospitalizations for index diagnosis^ββ^** | | | | |  |
|  | **12 months** | **1.230 (1.541)** | **1.374 (1.724)** | **0.908** (0.825-0.999)** | **0.903** (0.822-0.993)** |
|  | 30 days | 0.244 (0.484) | 0.249 (0.491) | 0.984 (0.844-1.146) | 0.977 (0.838-1.138) |
|  | 3 months | 0.561 (0.798) | 0.583 (0.823) | 0.979 (0.878-1.092) | 0.972 (0.873-1.083) |
|  | 6 months | 0.844 (1.116) | 0.901 (1.187) | 0.957 (0.865-1.059) | 0.950 (0.860-1.050) |
|  | 24 months (N=1,565) | 1.873 (2.336) | 1.896 (2.268) | 0.995 (0.884-1.119) | 0.924 (0.817-1.045) |
| **Mortality (any cause)^αα^** | | | | |  |
|  | **12 months** | **0.151 (0.358)** | **0.157 (0.364)** | **0.940 (0.808-1.097)** | **0.920 (0.792-1.071)** |
|  | 30 days | 0.038 (0.192) | 0.042 (0.200) | 0.936 (0.639-1.371) | 0.920 (0.630-1.343) |
|  | 3 months | 0.072 (0.258) | 0.072 (0.258) | 1.017 (0.771-1.343) | 0.999 (0.758-1.316) |
|  | 6 months | 0.098 (0.297) | 0.099 (0.299) | 1.011 (0.802-1.274) | 0.991 (0.788-1.246) |
|  | 24 months (N=1,565) | 0.275 (0.446) | 0.285 (0.452) | 0.980 (0.838-1.145) | 0.991 (0.788-1.246) |
| **Number of days of rehospitalization (LOS) for any cause** | | | | |  |
|  | **12 months** | **14.49 (24.32)** | **16.89 (28.98)** | **0.859**(0.750-0.985)** | **0.871**(0.760-0.998)** |
|  | 30 days | 3.301(8.138) | 3.873 (10.03) | 0.861 (0.746-1.046) | 0.861 (0.701-1.067) |
|  | 6 months | 9.932 (18.65) | 11.58 (22.23) | 0.863* (0.742-1.005) | 0.874* (0.749-1.018) |
|  | 24 months (N=1,565) | 19.11 (31.20) | 20.31 (34.85) | 0.940 (0.798-1.107) | 0.940 (0.798-1.107) |
| **Number of days of rehospitalization for index diagnoses** | | | | |  |
|  | **12 months** | **12.97 (22.56)** | **15.40 (27.43)** | **0.836** (0.725-0.964)** | **0.847** (0.734-0.976)** |
|  | 30 days | 3.053 (7.804) | 3.638 (9.921) | 0.840 (0.675-1.044) | 0.840 (0.677-1.042) |
|  | 6 months | 8.984 (17.24) | 10.59 (20.88) | 0.849** (0.724-0.994) | 0.853* (0.727-1.008) |
|  | 24 months (N=1,565) | 16.81 (29.35) | 18.26 (32.69) | 0.914 (0.769-1.086) | 0.921 (0.775-1.093) |
| **Health-related quality of life (EQ 5D-5L)^a^** ^δδ^ | | | | |  |
|  | at baseline | 0.747 (0.008) | 0.750 (0.009) | -0.003 (-0.028-0.021) | -0.002 (-0.026-0.021) |
|  | at 3 months | 0.640 (0.011) | 0.626 (0.011) | 0.010 (-0.018-0.039) | 0.007 (-0.023-0.038) |
|  | at 12 months | 0.600 (0.013) | 0.564 (0.012) | 0.032* (-0.003-0.066) | 0.034* (-0.001-0.069) |
| **Visits to GPs and specialists 12 months after discharge** | | | | |  |
|  | Patients with a GP visit^αα^ | 0.951 (0.215) | 0.962 (0.191) | 0.748 (0.509-1.100) | 0.758 (0.516-1.115) |
|  | Patients with a specialist visit^αα^ | 0.448 (0.498) | 0.449 (0.498) | 0.996 (0.850-1.167) | 0.998 (0.851-1.169) |
|  | Number of GP visits^ββ^ | 14.60 (9.484) | 14.54 (10.33) | 1.005 (0.954-1.058) | 1.005 (0.956-1.057) |
|  | Number of specialist visits^ββ^ | 1.163 (1.756) | 1.175 (1.912) | 0.993 (0.880-1.121) | 0.995 (0.882-1.123) |
|  | Continuity of care index (GPs)^δδ^ (N=2,406) | 0.683 (0.251) | 0.686 (0.252) | -0.004 (-0.024-0.016) | -0.003 (-0.023-0.0164) |
|  | Continuity of care index (GPs, cardiologists)^δδ^ (N=2,396) | 0.771 (0.251) | 0.770 (0.253) | -0.001 (-0.021-0.019) | 0.000 (-0.029-0.020) |
|  | Usual provider index (GPs) (N=2,406)^δδ^ | 0.771 (0.208) | 0.774 (0.209) | -0.004 (-0.020-0.013) | -0.003 (-0.0199-0.013) |
|  | Usual provider index (cardio.)^δδ^ (N=2,405) | 0.833 (0.204) | 0.833 (0.207) | -0.001 (-0.017-0.016) | 0.000 (-0.016-0.016) |
| **Adherence to medical advice^a^** ^αα^ | | | | |  |
|  | Medication unchanged after discharge (N=2,101) | 0.852 (0.355) | 0.795 (0.404) | 1.489*** (1.186-1.870) | 1.490*** (1.186-1.871) |
|  | Adherence to prevention recommendations (N=1,994) | 0.818 (0.386) | 0.738 (0.440) | 1.647*** (1.326-2.046) | 1.577*** (1.273-1.956) |
|  | Smoking cessation (N=1,079, excluding non-smoker) | 0.307 (0.462) | 0.306 (0.461) | 0.913 (0.694-1.201) | 0.960 (0.728-1.267) |
|  | Visited GP as recommended (N=1,994) | 0.946 (0.227) | 0.950 (0.218) | 0.769 (0.527-1.121) | 0.774 (0.531-1.130) |
|  | Visited a specialist as recommended (N=1,995) | 0.783 (0.412) | 0.711 (0.453) | 1.471*** (1.199-1.806) | 1.473*** (1.201-1.809) |

**Note:** Values for intervention and control group indicate the mean values. Standard deviations/errors in parentheses.

*** p<0.01, ** p<0.05, * p<0.1.

Model: αα Logistic regression, reported coefficients presented as odds ratios. ββ GLM, negative binomial distribution with log link, reported coefficients presented as incidence ratios. γγ Cox proportional hazards model, reported coefficients presented as hazard ratios. δδ OLS, robust;

Adherence to medical advice: assessed in phone call after 12 months (9–21 months in cases where 12-month call was not conducted) in the intervention group, control group after 12 months.
