# Supplementary material for: Reducing rehospitalization in cardiac patients: a randomized, controlled trial of a cardiac care management program (“Cardiolotse”) in Germany
Source: BMC Med. 2024 Oct 21;22:480. doi: 10.1186/s12916-024-03691-7 (PMC11492482; doi:10.1186/s12916-024-03691-7)
Supplement: Supplementary file 3 — Additional file 3: Table A2. Imputation values of the health-related quality of life (EQ-5D-5L) [file 12916_2024_3691_MOESM3_ESM.docx]

**Additional file 3**

Table A2: Imputation values of the health-related quality of life (EQ-5D-5L)

| **Health-related quality of life (EQ 5D-5L)** | | | | | | |  |
| --- | --- | --- | --- | --- | --- | --- | --- |
|  | | | | | | |  |
|  | **Intervention group** | | **Control group** | **Effect size OLS (95% CI)** | | **Adjusted effect size OLS (Age, gender, Charlson index) (95% CI)** | |
| **Mean values using a multiple imputation model** | | | | | | | |
| at baseline | | 0.747 (0.008)  N=1256 | 0.750 (0.009)  N=1294 | -0.003 (-0.028-0.022) | -0.003 (-0.028-0.021) | | |
| at 3 months | | 0.640 (0.011)  N=1256 | 0.626 (0.011)  N=1294 | 0.013 (-0.017-0.043) | 0.010 (-0.018-0.039) | | |
| at 12 months | | 0.600 (0.013)  N=1256 | 0.564 (0.012)  N=1294 | 0.036*(-0.000-0.072) | 0.032* (-0.003-0.066) | | |
|  | |  |  |  |  | | |
| **Mean values without imputations at all** | | | | | | | |
| at baseline | | 0.749 (0.085)  N=1092 | 0.753 (0.086)  N=1071 | -0.004 (-0.028-0.021) | -0.004 (-0.027-0.020) | | |
| at 3 months | | 0.706 (0.090)  N=919 | 0.692 (0.095)  N=951 | 0.014 (-0.014-0.041) | 0.009 (-0.018-0.037) | | |
| at 12 months | | 0.753 (0.063)  N=571 | 0.722 (0.074)  N=632 | 0.031** (0.016-0.061) | 0.038*** (0.009-0.068) | | |
|  | |  |  |  |  | | |
| **Mean values without imputation (only values for dead individuals imputed)** | | | | | | | |
| at baseline | | 0.749 (0.085)  N=1092 | 0.753 (0.086)  N=1071 | -0.004 (-0.028-0.021) | -0.004 (-0.027-0.020) | | |
| at 3 months | | 0.637 (0.125)  N=1018 | 0.625 (0.128)  N=1053 | 0.012 (-0.018-0.043) | 0.008 (-0.023-0.038) | | |
| at 12 months | | 0.559 (0.156)  N=770 | 0.541 (0.153)  N=770 | 0.018 (-0.020-0.056) | 0.023 (-0.014-0.061) | | |
|  | |  |  |  |  | | |
| **Mean values of complete case analysis** | | | | | | | |
| at baseline | | 0.777 (0.280) N=439 | 0.787 (0.284) N=448 | -0.009 (-0.046-0.028) | -0.005 (-0.040-0.031) | | |
| at 3 months | | 0.727 (0.287) N=439 | 0.714 (0.308) N=448 | 0.014 (-0.026-0.053) | 0.018 (-0.019-0.056) | | |
| at 12 months | | 0.761 (0.238) N=439 | 0.717 (0.277) N=448 | 0.044** (0.010-0.078) | 0.048*** (0.016-0.080) | | |

**Note:** Values for intervention and control group indicate the mean values. Standard deviations/errors in parentheses.

*** p<0.01, ** p<0.05, * p<0.1.
