# Supplementary material for: Reducing rehospitalization in cardiac patients: a randomized, controlled trial of a cardiac care management program (“Cardiolotse”) in Germany
Source: BMC Med. 2024 Oct 21;22:480. doi: 10.1186/s12916-024-03691-7 (PMC11492482; doi:10.1186/s12916-024-03691-7)
Supplement: Supplementary file 5 — Additional file 5: Figure A2. Time to Rehospitalization [file 12916_2024_3691_MOESM5_ESM.docx]

**Additional file 5**

Figure A2: Time to Rehospitalization
